# Quantification of tRNA fragments by electrochemical direct detection in small volume biofluid samples

**DOI:** 10.1038/s41598-020-64485-4

**Published:** 2020-05-05

**Authors:** Hazel McArdle, Marion C. Hogg, Sebastian Bauer, Felix Rosenow, Jochen H. M. Prehn, Kellie Adamson, David C. Henshall, Elaine Spain

**Affiliations:** 1 School of Chemical Sciences, National Centre for Sensor Research, Dublin City University, Dublin, D09 NR58 Ireland; 2 Department of Physiology and Medical Physics, Royal College of Surgeons in Ireland, 123 St. Stephen’s Green, Dublin, D02 YN77 Ireland; 3 FutureNeuro SFI Research Centre, Royal College of Surgeons in Ireland, 123 St. Stephen’s Green, Dublin, D02 YN77 Ireland; 4 Epilepsy Center Hessen, Department of Neurology, Baldingerstr, 35043 Marburg, Germany; 5 Epilepsy Center Frankfurt Rhine-Main, Neurocenter, Goethe-University, Schleusenweg 2-16, Haus 95, 60528 Frankfurt Germany; 6 LOEWE Center for Personalized Translational Epilepsy Research (CePTER), Frankfurt, Germany

**Keywords:** Molecular medicine, Biomarkers

## Abstract

Elevated levels of transfer RNA (tRNA) fragments were recently identified in plasma samples from people with epilepsy in advance of a seizure, indicting a potential novel class of circulating biomarker. Current methods for detection and quantitation of tRNA fragments (tRFs) include northern blotting, RNA sequencing or custom Taqman-based PCR assays. The development of a simple, at home or clinic-based test, would benefit from a simple and reliable method to detect the tRFs using small volumes of biofluids. Here we describe an electrochemical direct detection method based on electrocatalytic platinum nanoparticles to detect 3 specific tRFs: 5’AlaTGC, 5'GlyGCC, and 5'GluCTC. Using synthetic tRF mimics we showed this system was linear over 9 orders of magnitude with sub-attomolar limits of detection. Specificity was tested using naturally occurring mismatched tRF mimics. Finally, we quantified tRF levels in patient plasma and showed that our detection system recapitulates results obtained by qPCR. We have designed a tRF detection system with high sensitivity and specificity capable of quantifying tRFs in low volumes of plasma using benchtop apparatus. This is an important step in the development of a point-of-care device for quantifying tRFs in whole blood.

## Introduction

Epilepsy is one of the most common neurological disorders, affecting approximately 50 million people worldwide. Of those people, around one third do not respond to existing medications meaning they have to contend with unpredictable seizures that can impact daily living immensely. To date, much research into seizure prediction has focussed on analysis of electroencephalogram (EEG) data to look for rhythmicity in seizure patterns and enable algorithms to be designed to predict future seizure likelihood^1^. However, in a first-in-man study using implanted EEG electrodes, this approach only proved successful for some patients (approx. 2/3) whose seizures showed a detectable rhythmic pattern^2^. Blood-based molecules are attractive candidates for biomarkers of neurological conditions as samples can be relatively easily collected and small molecules that cross the blood-brain barrier can provide a read-out of underlying neuronal stress or damage. Studies to identify RNA biomarkers for epilepsy have focussed on microRNAs, reviewed in^3^. However, it is likely that other small noncoding RNAs may be potential biomarkers.

We recently identified fragments derived from transfer RNAs (tRNAs) as novel biomarkers in plasma from people with epilepsy, where we found them to be significantly elevated in advance of a seizure occurring^4^. tRNAs are one of the most abundant classes of RNAs in the cell, second only to ribosomal RNAs, which function in the same pathway^5^. Recent advances in RNA sequencing techniques and analysis led to the identification of short fragments derived from tRNAs in a range of different tissues and organisms^6^. tRNA cleavage is an active process rather than non-specific degradation, which indicates that tRFs may have acquired distinct functions from the full-length tRNA. tRNA cleavage occurs in response to a range of physiological stresses and the enzymes involved have homologs in Yeast indicating the pathway is highly conserved^7^. tRNA fragments (tRFs) can be derived from either the 5' or 3' end of the molecule and can range in size from ~18–30 nt fragments generated by cleavage in the D or T-loop, to tRNA halves (~32–44 nt) generated by cleavage within the anticodon loop. 3'tRNA fragments contain the CCA-tail indicating they originate from mature tRNA molecules.

We recently discovered that specific 5'tRNA fragments (5'tRFs) are elevated in plasma collected from people with epilepsy in advance of seizures and return to baseline levels rapidly post seizure^4^. This study suggests that plasma tRFs may be of use as a biomarker of seizure imminence, however in order for this to be of benefit to patients a point-of-care device capable of quantifying tRFs is required. In previous studies we have shown that direct detection of small noncoding RNAs, including miRNA-134, using platinum nanoparticles (PtNPs) resulted in attomolar detection limits in patient plasma samples and showed good correlation with Taqman-based qPCR quantification indicating this technique is suitable for low abundance RNA biomarker quantification^8^. Recent advances to this method led to the development of “TORNADO”, a theranostic one-step RNA detector capable of quantifying miRNA levels in unprocessed patient plasma and cerebrospinal fluid samples^9^. TORNADO is a centrifugal microfluidic device that is pre-loaded with the target miRNA, and platinum nanoparticles functionalised with probe miRNA. A three-electrode electrochemical cell is integrated into the device and by controlling the spin speed of the device, each of the chambers can be sequentially released and allowed to flow into the electrode chamber. The TORNADO device has been used to accurately quantify miRNA levels in plasma from people with mild cognitive impairment and Alzheimer’s Disease, which showed that miR-206 could predict cognitive decline^10^.

**Figure 1:**
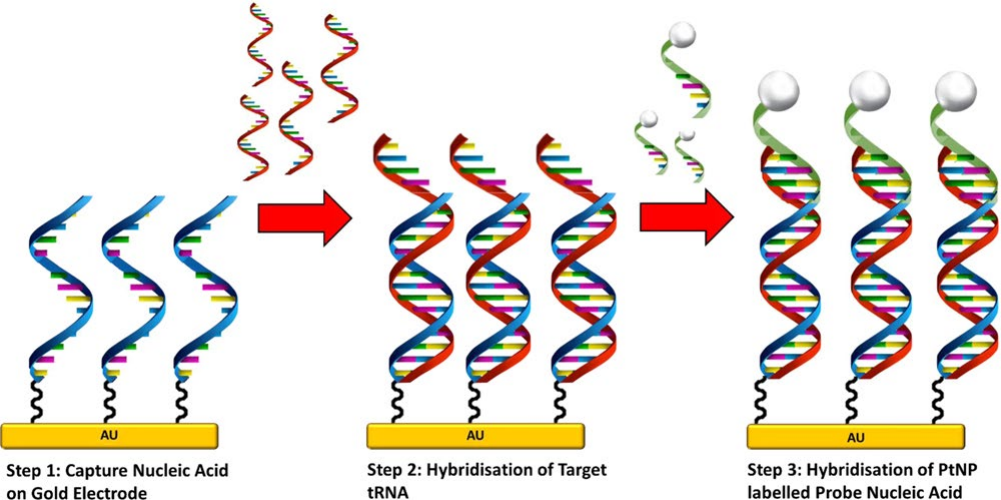
Schematic overview of sandwich assay formation on the gold disc electrodes.

Here we aimed to assess whether our amperometric detection method (Schematic overview in Fig. 1) can be modified to recognise tRFs, a new class of small non-coding RNA. Specifically, we tested whether three epilepsy-associated 5′tRFs can be detected in biofluids and replicate earlier findings that plasma tRF levels are elevated in advance of seizures.

## Results

### Quantification of tRFs by northern blotting

tRNAs are extensively modified and amplification-based approaches to quantify tRNAs can be skewed due to the inhibition of polymerase progression by heavily modified nucleotides. This can result in truncated reads, which stop at modified nucleotides and appear similar to reads obtained when tRNA cleavage has occurred. Therefore, it is vital to validate tRNA cleavage events by a non amplification-based approach such as northern blotting. Importantly, the amperometric quantification method described here does not require amplification and would therefore not be affected by modified nucleotides.

**Figure 2:**
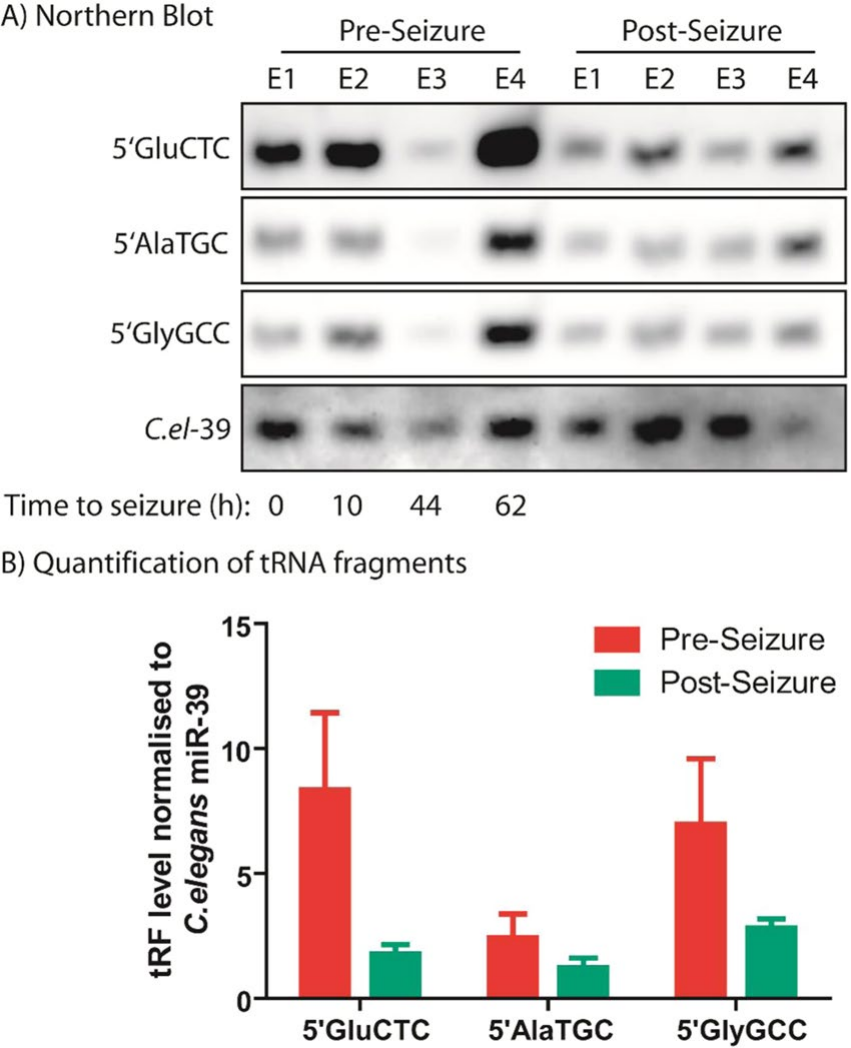
Northern blot quantification of tRFs in epilepsy patient plasma. (**A**) RNA extracted from plasma samples collected pre and post seizure from 4 epilepsy patients was analysed by northern blot. 20 ul total RNA was electrophoresed on a 15% gel and transferred to a nitrocellulose membrane and probed with dual digoxigenin-labelled probes for tRNA fragments and the *C.elegans* spike-in added during RNA purification. (**B**) Densitometric quantification of northern blot signals indicates all three tRNA fragments are higher in pre-seizure compared to post seizure samples.

To independently validate our original findings, we analysed RNA extracted from 4 patients (collected pre and post seizure) by northern blot and quantified signals by densitometry (Fig. 2). Specific probes to recognise the 5′tRFs (5′GluCTC, 5′AlaTGC, and 5′GlyGCC) were used along with a probe to recognise the *C.elegans* miRNA-39 spike-in that was added during purification. The probes are labelled with digoxigenin conjugated to the 5′ and 3′ ends and were used at a final concentration of 1 nM. Pre-seizure samples were collected over a range of 0–62 hours before a seizure occurred, whilst post-seizure samples were collected 24 hours after a clinically confirmed seizure had occurred. Here we could show elevated levels of all 3 tRFs in samples collected pre-seizure compared to post-seizure samples.

### Electrocatalytic detection of tRFs in clean buffer

Platinum nanoparticles are highly electrocatalytic for the reduction of hydrogen peroxide. In this  study, platinum nanoparticles are confined to the surface of a gold disc electrode via complementary nucleic acid hybridisation. When hydrogen peroxide is injected into the electrochemical cell, the platinum nanoparticles electrocatalyse the reduction of hydrogen peroxide, generating a current that is directly proportional to the number of nanoparticles on the electrode surface. The number of nanoparticles on the electrode surface depends on the concentration of target nucleic acid. Therefore, the concentration of target nucleic acid directly correlates to the current generated. To analyse our tRF detection method we first used synthetic tRF mimics that were made from DNA and hybridised in Denhardt’s buffer solution.

**Figure 3:**
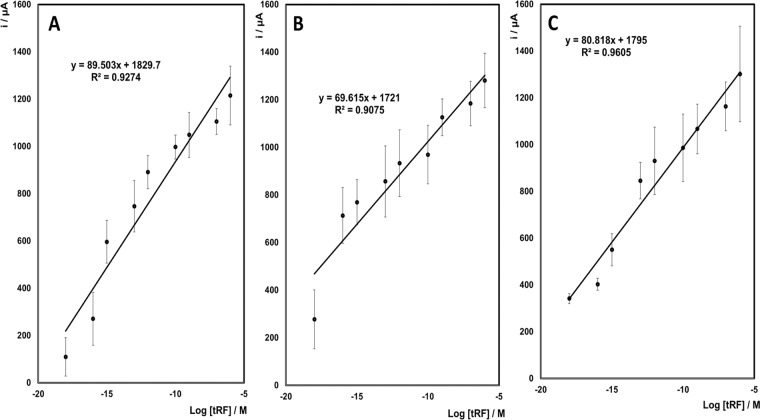
Linear current response to log [tRF]. Dependence of the difference in current before and after the addition of H_2_O_2_ on log [tRFs] for a 2 mm diameter gold disc electrode following hybridisation with probe tRFs that are labelled with platinum nanoparticles. The applied potential is −0.25 V in 0.1 M H_2_SO_4_. Δi represents the difference in current before and after the addition of 2 mM H_2_O_2_. Error bars indicate standard deviation from n = 3 replicates. (**A**)**:** target: 5’AlaTGC; (**B**)**:** target: 5’GluCTC; (**C**)**:** target: 5’GlyGCC. Raw data are provided in Supplementary Table 1.

A fixed potential of −0.25 V was applied to the working electrode, and the difference in current between the absence of hydrogen peroxide and the addition of 2 mM hydrogen peroxide was measured. Figure 3 shows the dependence of the change in current (Δi) on the concentration of the tRF (log[tRFs]) using varying concentrations of the three 5′tRFs in clean buffer. Raw data for calibration plots shown in Figure 3 are provided in supplementary Table 1. Looking at the 5′AlaTGC tRNA detection assay (Fig. 3A), the best-fit least-squares regression line equation is y = 89 ± 10×+ 1830 ± 117, with a relative standard deviation (RSD) of between 4–40% (n = 3) for all concentrations. The sensitivity of the assay (slope of calibration curve) is high, with a wide dynamic range over 9 orders of magnitude, and the curve is linear between 1 aM and 100 nM, with an R^2^ value of 0.9274. These results indicate a high binding affinity of the target tRF to the capture strands on the modified electrode surface.

Figure 3B shows the calibration curve for the 5′GluCTC tRNA detection assay. The best-fit least-squares regression line equation is y = 69.6 ± 8×+ 1721 ± 104, with an RSD of between 7–17% (n = 3) for all concentrations. The curve is linear between 100 aM and 1 µM, with an R^2^ value of 0.9075. This curve has an equally high sensitivity with a dynamic range over 9 orders of magnitude.

Figure 3C is the calibration plot for the final tRF looked at in this study, 5′GlyGCC. The equation for the best-fit least-squares regression line for this target is y = 81 ± 6×+ 1795 ± 77, with an RSD of between 6–16% (n = 3) for all concentrations. The dynamic range is over 10 orders of magnitude with high sensitivity and a linear range from 1 aM to 1 uM, with an R^2^ value of 0.9605.

The limit of detection (LOD) is determined by utilising both the measured Limit of Blank (LOB) and test replicates of a sample known to contain a low concentration of analyte, using the following equation:

1 $${\rm{LOD}}={\rm{LOB}}+1.645({\rm{Std}}.\,{\rm{Dev}}.\,{\rm{Low}}\,{\rm{conc}}\,{\rm{sample}})$$

The LOD is defined as the lowest analyte concentration likely to be reliable from the blank. The LOD for 5′AlaTGC is calculated to be 0.12 aM, for 5′GluCTC is calculated to be 0.17 aM, and LOD for the 5′GlyGCC detection is calculated to be 0.17 zM. While the ability of the assay to detect these concentrations has not been demonstrated, it is estimated that detection is feasible to these concentrations of target analyte. The wide dynamic range, approximately 9 orders of magnitude, low LOD, and excellent sensitivity make these assays to detect novel tRF biomarkers attractive for the development of a label-free, point-of-care diagnostic device.

### Specificity of tRF detection system

**Figure 4:**
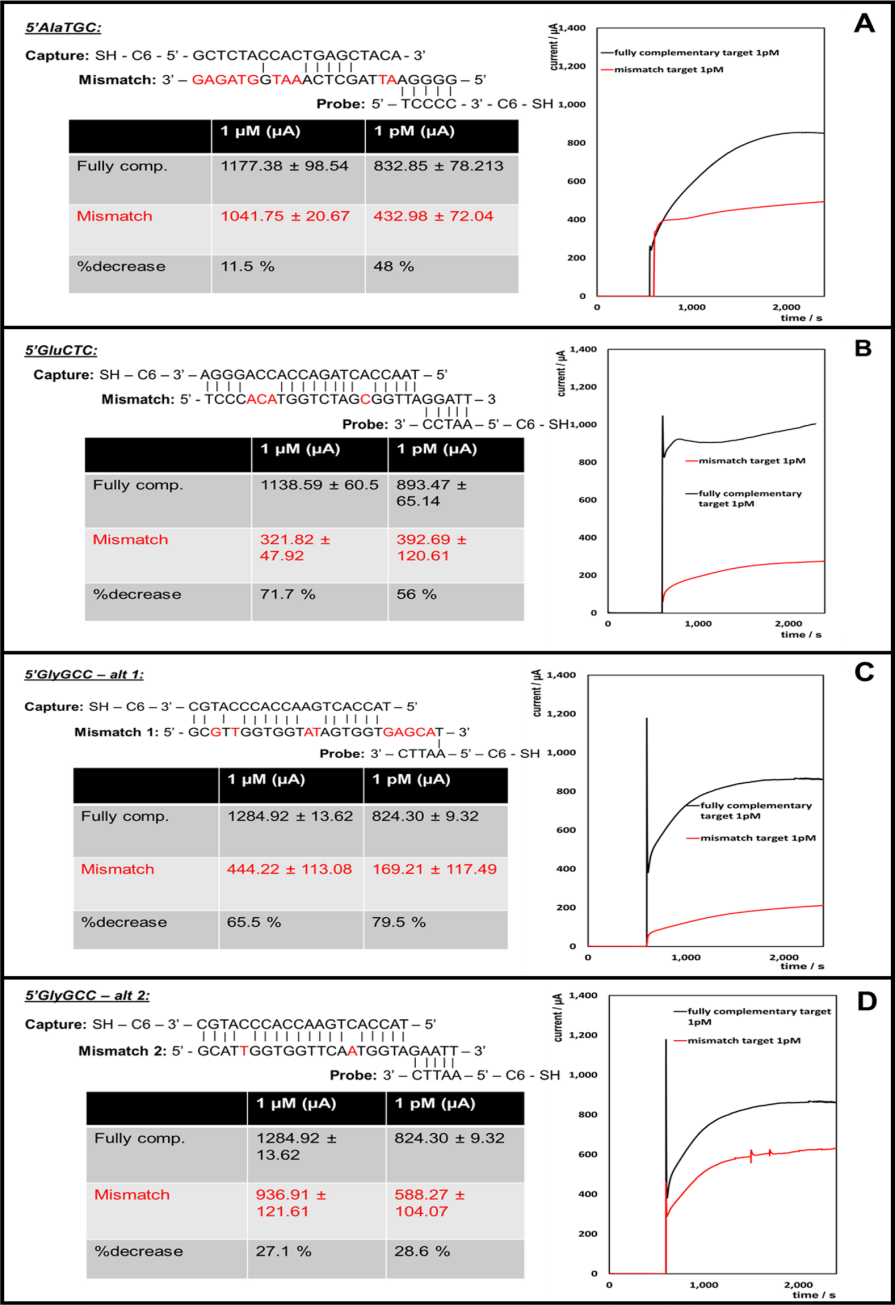
Mismatches reduce the signal indicating detection is sequence specific. Amperometric i-t curves showing the current difference between a fully complementary strand (black line) and the base mismatch strand (red line). The tables show the current value of each nucleic acid strand, and the percentage decrease. The nucleic acid sequences of the mismatches for each target are shown also. (**A**) Target: 5′AlaTGC; (**B**) target: 5′GluCTC; (**C**) target: 5′GlyGCC, mismatch 1; (**D**) target: 5′GlyGCC, mismatch 2.

To determine the specificity of the detection system synthetic tRFs sequences were used that occur naturally but contain several mismatches compared to the tRFs of interest. Targets containing mismatches of between 2–11 base mismatches were selected. Figure 4 shows the mismatches and how they impact on the detection system. It also indicates the percentage decrease in current between the fully complementary target and the mismatched target strands. Amperometric i-t curves comparing the fully complementary to the mismatch target at a certain concentration are presented; this clearly shows how the current generated at the electrodes is significantly decreased for each of the mismatches.

For the 5′AlaTGC mismatch (Fig. 4A), an extra base is present in the tRF, which causes a total of 11-base mismatches. At  1 µM target concentration, there is an 11.5% decrease in current and for the 1 pM target concentration there is a 48% decrease in current generated. Initially it was thought that this is a relatively small drop in current, however, when the positions of the mismatches were examined, it was determined that the target strand could still attach to the capture strand as there are many consecutive complementary base pairings towards the end of the capture strand. If the target strand is hybridised to the capture, then the platinum nanoparticle labelled probe strand would be brought to the surface of the electrode also, as all of the probe strand is complementary. This accounts for the relatively large current generated for the 5′AlaTGC mismatch.

For the 5′GluCTC alternative (Fig. 4B), there is a 4-base mismatch; this causes a 55–70% current decrease from the fully complimentary strands. This indicates once again that this detection system can differentiate well between the fully complementary target and other naturally occurring tRF strands, and that the current generated is in response to the 3 tRFs that are being investigated. It has been previously demonstrated in similar assays that the electrocatalytic current decreases by approximately a factor of 4 in the presence of 1 base mismatch^11,12^, which further confirms the specificity of this sensor. Additionally, the electrocatalytic reaction at the platinum nanoparticles causes any non-specifically bound RNAs to be desorbed from the nanoparticle surface, making the sensor response insensitive to non-specific binding.

For the 5′GlyGCC tRF, two different tRF mismatches were tested; one which had a 9-base mismatch (Alt 1), and one which had a 2 base mismatch (Alt 2). Alt 1 has a current decrease of 65–80%, due to the large number of base mismatches. As none of the bases present on the probe strand are complimentary to the target strand, even if the target strand hybridises, the probe will not; therefore, a high current cannot be generated. Alt 2 has a current decrease of approximately 30% for both concentrations. Due to the position of the two mismatches, some of the target strand is likely to still hybridise to the capture, which in turn will bring the probe strand to the surface, resulting in the current generation.

### Quantification of tRF levels in plasma collected from people with Epilepsy

**Figure 5:**
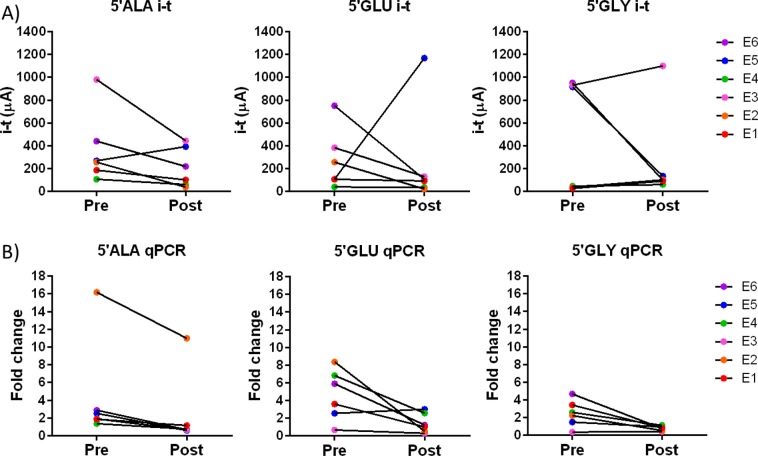
Plasma tRF levels quantified by amperometric analysis and qPCR. (**A**) Amperometric and (**B**) TaqMan qPCR based quantification of tRNA fragment levels in plasma collected from epilepsy patients pre and post seizure. Amperometric analysis was performed on whole plasma samples whereas total RNA was extracted prior to TaqMan analysis of tRNA fragment levels. Data for qPCR is from a subset of patients that were previously published^4^. Epilepsy patients (E1-E6) are colour coded with the same colours in all graphs.

Analysing synthetic tRFs in buffer allowed us to assess the specificity and range of the tRF quantification system, however biofluid samples can contain many contaminants such as other RNA species or proteins, which may inhibit detection of the desired target. Therefore, the ultimate assessment of this device is to determine whether it can detect a difference in tRF levels in plasma samples collected pre and post seizure from the same patient. Here we analysed plasma from 6 patients who were included in the original study, with pre-seizure samples collected between 0–62 hours before seizure onset occurred. Post seizure samples were collected 24 hours after an electro-clinically confirmed seizure (or seizures) had occurred. Four of these patient samples were also assessed by northern blotting (Fig. 2), which confirmed that the tRFs were present in these samples. Here we found that tRFs were detected and quantifiable in all samples (Fig. 5B, Table 1). In the majority of samples, the tRF levels were higher in pre-seizure samples. The 5′GlyGCC assay appears to show an all-or-nothing signal with little sensitivity to subtle changes in tRF levels, indicating this assay may not be suitable for a seizure prediction device. However, 5′AlaTGC and 5′GluCTC levels show similar fold-change between pre and post seizure samples indicating these would be best for further development. The absolute concentrations of the 3 tRFs in plasma samples, calculated using the standard curves shown in Fig. 3, are provided in Table 1. In the majority of samples, the tRF levels were higher in pre-seizure samples, however in some cases the levels were below the estimated LODs calculated earlier. This was more common in post-seizure samples, which are less clinically relevant as we aim to develop a system to detect pre-seizure increases in tRFs.

**Table 1:** Absolute quantification of tRF levels in plasma samples from epilepsy patients.

5′AlaTGC	Patient	Molarity	5′GluCTC	Patient	Molarity	5′GlyGCC	Patient	Molarity
Pre-seizure	E1	0.44aM	Pre-seizure	E1	0.01 zM	Pre-seizure	E1	0.13 zM
	E2	2.65 aM		E2	0.9 zM		E2	0.17 zM
	E3	324.02 pM		E3	0.06 zM		E3	19.81 pM
	E4	0.06 aM		E4	0.01 zM		E4	0.23 zM
	E5	3.67 aM		E5	0.01 zM		E5	13.07 pM
	E6	0.41 aM		E6	11.75 fM		E6	36.66 pM
Post-seizure	E1	0.05 aM	Post-seizure	E1	0.01 zM	Post-seizure	E1	0.80 zM
	E2	9.22 zM		E2	0.01 zM		E2	1.08 zM
	E3	328.27 aM		E3	0.01 zM		E3	2.49 fM
	E4	0.02 aM		E4	0.01 zM		E4	0.34 aM
	E5	86.85 aM		E5	27.82 aM		E5	2.83 zM
	E6	0.99 aM		E6	0.01 zM		E6	1.03 zM

Changes in current were used to calculate absolute levels of tRFs using the curves shown in Fig. 3. The majority of pre-seizure samples showed levels above the LOD however some post seizure samples were below the estimated LODs.

### Comparison of detection methods

The epilepsy patient samples analysed in this study were previously assessed for tRF levels using custom TaqMan assays^4^. To determine whether the amperometric quantification method can detect the same difference in tRF levels we compared the data from the two methods. Comparison of amperometric and TaqMan qPCR quantification methods reveals that the amperometric detection method validated earlier findings showing a difference in tRF levels in advance of a seizure compared to samples collected post seizure from the same patients (Fig. 5A,B). Additionally, the range of signals detected was similar for the two quantification methods indicating over 4-fold higher signals in pre-seizure samples compared to post seizure samples for both quantification methods. The qPCR method showed a greater spread of signals as this method uses a logarithmic calculation which can amplify small changes. The 5'AlaTGC and 5'GluCTC assays showed the best correlation with PCR-based quantification methods indicating these tRF quantification assays would be the best for future studies.

## Discussion

The ability to predict the onset of epileptic seizures could transform the daily lives of millions of people living with drug-resistant epilepsy who experience unprovoked seizures with no warning. We recently identified three tRFs that are elevated in advance of seizures occurring in plasma from people with epilepsy indicating they may be of use as biomarkers of seizure imminence^4^. In that study, custom Taqman assays were used to allow high throughput quantification of tRFs in patient plasma samples. However, for this discovery to be of use for people with epilepsy we require a different method of detection as PCR based quantification requires standard laboratory equipment and, although relatively fast, would not be feasible for a daily monitoring system. Direct detection of RNA molecules in biofluids would enable point-of-care monitoring of tRF levels. There have been many electrochemical-based biosensors for the detection of nucleic acids developed in recent times; they are ideally suited for point-of-care diagnostics, as they are sensitive, simple and inexpensive^13^. We have demonstrated that our method shows high sensitivity, with significant decreases in signal when using mismatched synthetic tRF mimics. However, the difference in detection was small where the mismatch was created by insertion of an additional base. This is likely due to the remaining complementarity between regions either side of the insertion. The location of mismatches also appears to influence the difference in detection signal, with a better difference in signal when the mismatches are located in the probe region; this is due to the electrocatalytic response signal being generated by the platinum nanoparticle, which cannot be immobilised on the surface without the complementary probe strand. One potential way to overcome these issues would be to include all three capture strands in one single device. We have found good correlation between levels of the three tRFs investigated here, such that when one tRF is high, the others are also elevated (see northern blot data in Fig. 2). This suggests these three tRFs are generated as part of the same stress response, and a method to detect a combination of all three tRFs may provide enhanced sensitivity and specificity. This could be achieved by using a single microfluidic device that includes surfaces modified by the three corresponding capture strands; this would ensure that a response is achieved when any of the tRFs are elevated. Azimzadeh *et al*., have recently reported an electrochemical sensor for the detection of miR-155, which is associated with breast cancer, using differential pulse voltammetry^14^. They have used graphene oxide, gold nanorods, and a novel electrochemical indicator, and have achieved similar turnaround times to the system detailed here; however, the limit of detection reached is not as low as the LOD reported here at 0.6 fM. Guo *et al*., have reported a limit of detection of 15 aM for their miR-196a biosensor^15^, however their linear range is not as extensive as ours and the method is complex and time consuming. Additionally, they have not validated their detector using patient samples; the data are from spiked plasma samples. In this study, we first tested our method using synthetic tRF mimics and then used the method to analyse patient plasma samples to validate our method.

In our protocol described here, the electrolyte used contains 2 mM H_2_O_2_ in aqueous 0.1 M H_2_SO_4_. In our previous study DPBS is used, along with 20 µM H_2_O_2_^9^; this is due to the measurements being carried out inside a microfluidic device, which eliminates the need for sulfuric acid. Studies carried out by Sowmya *et al*., 2013, showed that DNA was obtainable after the first 2 hours of concentrated H_2_SO_4_ immersion and there was an inverse proportional relationship between mean absorbance ratio and quantity of obtained DNA on an hourly basis^16^. Our focus is firmly on single shot sensors suitable for use with blood samples, making regeneration of the sensor of minor importance. Using DPBS is ideal and would be the next step in the development of a point-of-care device for the detection of the tRFs discussed in this manuscript. A lower concentration of H_2_O_2_ is sufficient here, as the microfluidic device uses microscale volumes, whereas on standard electrochemical cells, these are macroscale. The use of highly electrocatalytic nanoparticles that generate an easily measured current even for low surface coverages means that little reagent is needed, reducing cost and reducing the need to regenerate the sensor i.e., this detection strategy is for single use. Additionally, while non-specific adsorption can be an issue for some sensors, we don’t believe that it is an issue in this instance. We have demonstrated previously that when there is no target present no electrocatalytic signal is observed^8,9,17^. This indicates non-specific binding is not an issue in this sandwich assay. One potential issue with highly catalytic PtNPs is that they may damage DNA or disrupt the target before detection. However, we did not encounter this problem during our experiments; the current signal generated stabilises within approximately ten minutes of adding the hydrogen peroxide (as shown in the i-t curves in Fig. 4). The current signal is read after 20–30 minutes, to ensure complete stabilisation has occurred. The nucleic acid strands are thiol terminated and this is how they attach to the platinum nanoparticles. Any non-specific binding to the PtNPs are desorbed during the electrocatalytic reaction, however the thiol-bonded nucleic acids are stable on the surface.

One of the reported major drawbacks to electrochemical biosensors is the inability to be made compact, portable and point-of-care^18^. As we have shown previously^9^, we have integrated our electrochemical nucleic acid detection method into a portable microfluidic device. The current method utilises benchtop equipment and could be incorporated in a hospital environment, however for this assay to be of use as a continuous monitoring system a portable device would be required. We envisage a device similar to a glucose monitor currently widely used by people with diabetes to monitor blood glucose daily in any environment. Such devices require a pinprick of whole blood. Further experiments are required, therefore, to determine whether tRFs can be detected a) in whole blood, and b) in a pin-prick volume. Plasma equals approximately 60% of the total volume of whole blood. Here we showed that our method can accurately quantify tRFs in low volume (30 µl) plasma samples. This suggests our method should be able to accurately quantify tRFs in a finger-prick (~50 µl) volume. However, further work is required to determine whether our method is accurate using whole blood, as there may be inhibitory or interfering factors present.

Another potential drawback to this current method is the long hybridisation times. As mentioned, the TORNADO device is a possible prototype for further development of tRF detection. When using centrifugal microfluidics, a major advantage is the ability to control the rotation speed, which is turn controls the flow of liquid in the device. Due to the design of the microchannels in the device, the hybridisation time is lower than when carrying out static incubation. This is due to the diffusion distance inside the device being much shorter than when at macroscale, which accelerates the hybridisation kinetics. Therefore, the overall time to perform the sandwich assay on the sensor surface will be drastically shortened. This study provides the first stage in development of such a device, showing that tRFs can be quantified using a direct detection method in low volume plasma samples from people with epilepsy, and that our new method replicates earlier findings that showed a difference in tRF levels in samples collected before compared to after a seizure has occurred.

Validation of our method in epilepsy patient plasma samples provides valuable data on the applicability of this method for clinical use. In comparison to the qPCR-based approach the direct detection method showed good correlation however the range was not as extensive. This is likely due to the logarithmic scale used to analyse the qPCR fold-change which amplifies small differences in signal. The 5′AlaTGC and 5′GluCTC direct detection methods showed broad overall correlation between methods. The 5′GlyGCC direct detection in patient plasma samples showed poor correlation with the Taqman-based qPCR data for 3 out of 6 patient samples analysed (Fig. 5). This could indicate that the 5′GlyGCC detection shows an all-or-nothing response and may lack the sensitivity required to detect subtle changes in tRF levels. However, GlyGCC is one of the most abundant tRFs present in biofluids so this result could indicate similar fragments are present and contributing to the signals detected by this method. Inter-ictal and seizure activity can display rhythmic patterns in some patients, following ultradian, circadian, or multidien rhythms, reviewed in^19^. However, it remains to be seen whether circadian rhythm can influence circulating tRF levels.

Some of the post-seizure samples are below the estimated LODs, however, as we are primarily interested in the rise in the tRFs in pre-seizure samples, this is not of major concern. A small number of the pre-seizure plasma samples give a signal below the estimated LOD. We believe this may be due to the low volume (30 µL), as the calibration data was performed using 200 µL of target nucleic acid, and potential inhibitory factors from the plasma. One potential method for increasing the signal is to use regioselectively functionalised platinum nanoparticles. Previously, we have demonstrated the synthesis of these nanoparticles and their function in a sandwich assay^11^. These PtNPs are hemispherical and have a dual function; one side has probe strand nucleic acids immobilised on the surface, and the flat underside is clean for electrocatalysis of the peroxide. This gives a larger surface area for the catalysis to occur and therefore gives a larger signal (up to 3 mA). Using these, in place of the uniformly functionalised spherical PtNPs, a larger signal may be achieved for the tRFs investigated here. Additionally, we propose to extend the range of the calibration curve below attomole level, to more accurately assess the limits of our sensor.

tRFs are currently under investigation as biomarkers for neurological disease and many different cancers indicating a point-of-care tRF quantification device could have broad application either in a hospital-based setting or with further development as an at-home device^4,20^. The role of tRFs in circulation has not been extensively studied yet it is thought they may act as signalling molecules. In cell-based and animal studies tRFs have been associated with a range of functions including stress granule formation^21^, inhibition of protein translation^22^, inhibition of cytochrome c signalling^23^, regulation of ribosome biogenesis^24^, epigenetic transmission of metabolic defects in sperm^25,26^, and regulation of proliferation and metastasis in cancers^27,28^. Further studies are required to determine the function of tRFs in circulation, and their role in epilepsy, but they remain attractive candidates for development of a seizure prediction monitoring device. To the best of our knowledge, label-free electrochemical detection of tRFs has not yet been reported. We demonstrate a rapid, accurate alternative to current methods, with the potential ability to be miniaturised for portable, point-of-care diagnostics.

## Conclusions

Here, a novel class of circulating biomarkers that are implicated in neurological disorders and cancers are directly detected with using benchtop apparatus. Our method can reliably quantify sub-attomolar tRF levels, which demonstrates it detects clinically relevant range of tRFs in circulation. Currently, approximately 30% of the patient samples were below the limits of detection, however we believe that this method can be significantly improved by enhancing the LOD further, making this direct detection method useful for clinical applications. The next steps would be to incorporate this detection system into a microfluidic device, similar to TORNADO^9^, modified with the ability to simultaneously detect the three tRFs in patient plasma samples. We envision to further improve the system by applying the detection method to whole blood samples, with the end goal being point-of-care detection of the tRFs in a pinprick of blood, to predict a seizure in patients with epilepsy.

## Methods

### Materials

Denhardt’s Hybridisation solution (≥99.5%) for RNA strand assembly was used as received from Sigma Aldrich. Platinum nanoparticles (50–70 nm) were purchased from Strem Chemicals. Hydrogen Peroxide (30% w/v) was used as received from Sigma Aldrich. All aqueous solutions were prepared using RNase free water. The oligonucleotides were purchased from Eurogentec with a purity of >98%. The base sequences are listed in Table 2.

**Table 2:** Oligonucleotides for tRNA fragment detection. Mismatched bases are underlined and in bold.

**5**′**AlaTGC:**	
Target:	5′–GGGGATGTAGCTCAGTGGTAGAGC-3′
Capture:	SH - C6–5′-GCTCTACCACTGAGCTACA-3′
Probe:	5′–TCCCC - 3′ - C6 - SH
Mismatch:	5′-GGG GA**A T**TA GCT CA**A AT**G **GTA GA**–3′
**5**′**GlyGCC:**	
Target:	5′–GCATGGGTGGTTCAGTGGTAGAATT-3′
Capture:	5′-TACCACTGAACCACCCATGC-3′ - C6 - SH
Probe:	SH - C6–5′–AATTC-3′
Mismatch 1:	5′-GC**G** T**T**G GTG GT**A T**AG TGG T**GA GCA** T–3′
Mismatch 2:	5′-GCA T**T**G GTG GTT CA**A** TGG TAG AAT T–3′
**5**′**GluCTC:**	
Target:	5′–TCCCTGGTGGTCTAGTGGTTAGGATT-3'
Capture:	5′–TAACCACTAGACCACCAGGGA-3′- C6 - SH
Probe:	SH - C6–5′-AATCC-3′
Mismatch:	5′-TCC C**AC A**TG GTC TAG **C**GG TTA GGA TT–3′

#### Study approval

Samples were collected at the Philipps University of Marburg in Marburg, Germany. Ethical approval was obtained from the Marburg medical ethics committee (MAR, 17/14), written informed consent was obtained from all participants according to Declaration of Helsinki principles, and all experiments were performed in accordance with the guidelines.

#### Epilepsy patient samples

A 10 ml blood sample (pre-seizure) was taken on admission. A post-seizure sample was collected 24 h after experiencing an electro-clinical seizure documented by video-EEG monitoring. The interval between pre-seizure blood sampling and seizure occurrence varied among patients, as did the number and type of seizures experienced.

### Plasma preparation

Blood was collected by venupuncture into a K2-EDTA tube, gently inverted several times and processed into plasma within 1 hour of collection. Plasma was prepared by centrifuging tubes at 1300 x g for 10 minutes at 4 °C. A second centrifugation step was performed at 1940 x g for 10 minutes at 4 °C to reduce cellular contamination. Samples were stored at −80 °C.

### RNA extraction

RNA was purified from 200 ul plasma using the Qiagen serum/plasma miRNeasy kit (Qiagen). *C.elegans* miRNA-39 spike-in (at 1.6 × 10^8^ copies/ul) was added during purification according to the manufacturers instructions (Qiagen). RNA was eluted in 40 ul water containing 1 ul RNaseOUT ribonuclease inhibitor. 2 ul total RNA was used per Taqman assay and 20 ul total RNA was loaded onto a gel for northern blotting.

### qPCR

Custom small RNA Taqman assays (ThermoFisher Scientific) were designed to tiRNA fragments and quantification was performed on a 384-well Quantstudio 5 PCR machine (ThermoFisher Scientific). tRF levels were normalised to *C.elegans* miRNA-39 spike-in, using the 2^−DDCt^ method^29^. 2 ul RNA was used per reverse transcription which as performed according to Taqman small RNA Assay protocol (ThermoFisher). 1 ul of the reverse transcription was used in the qPCR reaction, and qPCRs were performed in triplicate.

### Northern blotting

Total RNA was diluted in formamide loading buffer and denatured at 90 °C for 5 minutes before loading onto a 15% TBE-Urea PAGE gel. Gels were electrophoresed at 200 V for 95 minutes at 4 °C . Northern blotting was performed as described^30^. Briefly, following gel electrophoeresis RNA was transferred to a Hybond+ nitrocellulose membrane (GE Lifesciences) at 10 V for 60 minutes at 4 °C. RNA was UV-cross-linked to the membrane in a Stratalinker (1200 mJ/cm2). Membranes were blocked in ultraHyb Oligo (ThermoFisher Scientific) for 30 minutes at 37 °C. Dual digoxigenin labelled DNA probes were heat denatured at 95 °C, added to blocking solution (final concentration 1 nM), and incubated overnight at 37 °C. Membranes were washed twice with low stringency wash buffer (2x SSC, 0.1% SDS) and twice with high stringency wash buffer (0.1x SSC, 0.1% SDS) at 37 °C then washed in 2x SSC at room temperature. Membranes were then processed using the DIG wash and block set according to the manufacturer’s instructions (Roche). CPD-Star Development Reagent (Roche) was added to the membrane and images were acquired using an LAS 4000 Reader (Fujifilm). Dual digoxigenin labelled DNA probes used are listed in Table 3.

**Table 3:** Dual digoxigenin-labelled probes used for northern blotting.

Name	Sequence
5′AlaTGC	5′-GCTCTACCACTGAGCTACATCCCC-3′
5′GluCTC	5′-AATCCTAACCACTAGACCACCAGGGA-3′
5′GlyGCC	5′-AATTCTACCACTGAACCACCAATGC-3′

### Sandwich assay formation on electrode surface

Figure 1 illustrates the amperometric tRF detection method, showing how the sandwich assay was formed on the electrode surface. A monolayer of capture strand DNA was formed on the surface of a freshly polished and electrochemically cleaned 2 mm gold disc electrode by immersing in 200 µL of a 1 µM solution of the capture strand dissolved in Denhardt’s buffer. This capture DNA strand is complementary in part to the target tRFs. After 5 hours, the electrode was rinsed with RNase-free water to remove any loosely bound strands. Hybridisation of the target strand to the immobilised capture strand was carried out at 37°C for 3 hours. Different concentrations of the target ranging from 1 aM to 1 µM were used in 200 µL of buffer. Following hybridisation, the electrode was rinsed thoroughly with RNase-free water.

Platinum nanoparticles were functionalised with a probe nucleic acid strand, which is complementary to part of the target tRF, by incubation overnight at 37°C. These labelled nanoparticles were brought to the surface of the electrode via complementary hybridisation. The nanoparticle labelled probe DNA is complementary to the section of the target that was not used for hybridising to the capture strand, and the sandwich assay is completed by incubating the modified electrode with the probe labelled platinum nanoparticles for 4 hours at 37 °C in a volume of 200 µL. Before quantification, the electrode was thoroughly washed with RNase-free water.

### Amperometric detection

A three-electrode electrochemical cell was used; the working electrode was a 2 mm gold disc electrode, the counter electrode was a large area coiled platinum wire and a silver/silver chloride (Ag/AgCl in 3 M KCl) acted as the reference electrode. All electrochemistry experiments were carried out on a CHI650 workstation, with 0.1 M H_2_SO_4_ as the supporting electrolyte.

A constant potential of −0.25 V was applied to the electrode and the resulting current was measured. After 10 minutes, the current reached an equilibrium and sufficient hydrogen peroxide was added to give a final concentration of 2 mM. The current associated with the reduction of the peroxide at the bound platinum nanoparticles was measured at equilibrium after 30 minutes. The analytical response is taken as the difference in current, measured before and after the addition of the hydrogen peroxide.

## Supplementary information

Supplementary Data File.
