# Supplementary material for: Quantification of tRNA fragments by electrochemical direct detection in small volume biofluid samples
Source: Sci Rep. 2020 May 5;10:7516. doi: 10.1038/s41598-020-64485-4 (PMC7200677; doi:10.1038/s41598-020-64485-4)

**Supplementary data for manuscript:**

**Quantification of tRNA fragments by electrochemical direct detection in small volume biofluid samples**

Hazel McArdle^1^*^, Marion C. Hogg^2,3^*^, Sebastian Bauer^4,5,6^, Felix Rosenow^4,5,6^, Jochen H.M. Prehn^2,3^, Kellie Adamson^1^, David C. Henshall^2,3^, and Elaine Spain^1^.

**Working title: tRNA fragment detection manuscript**

1. School of Chemical Sciences, National Centre for Sensor Research, Dublin City University, Dublin 9, Ireland.

2. Department of Physiology and Medical Physics, Royal College of Surgeons In Ireland, St. Stephen’s Green, Dublin D02 YN77, Ireland.

3. FutureNeuro Research Centre, Royal College of Surgeons In Ireland, St. Stephen’s Green, Dublin D02 YN77, Ireland.

4. Epilepsy Center Hessen, Department of Neurology, Baldingerstr, 35043, Marburg, Germany.

5. Epilepsy Center Frankfurt Rhine-Main, Neurocenter, Goethe-University, Schleusenweg 2-16, Haus 95, 60528, Frankfurt, Germany

6. LOEWE Center for Personalized Translational Epilepsy Research (CePTER), Frankfurt, Germany.

* Correspondence: hazel.mcardle@dcu.ie or marionhogg@rcsi.ie

^ These authors contributed equally to this work.


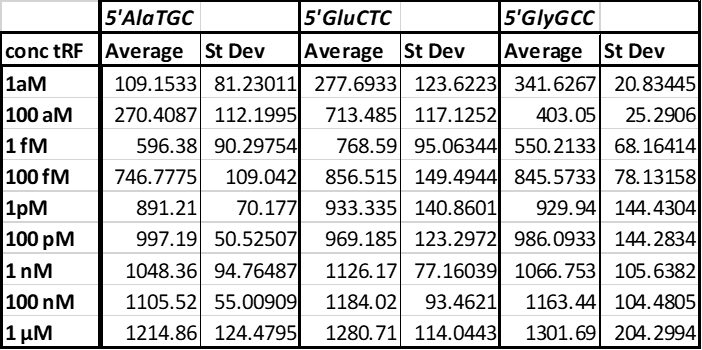


**Table 1: Raw data used in to generate graphs shown in Figure 3.** Where average and standard deviation (St Dev) of n = 3 replicates is shown.

Original blots for northern blot shown in Figure 2 A


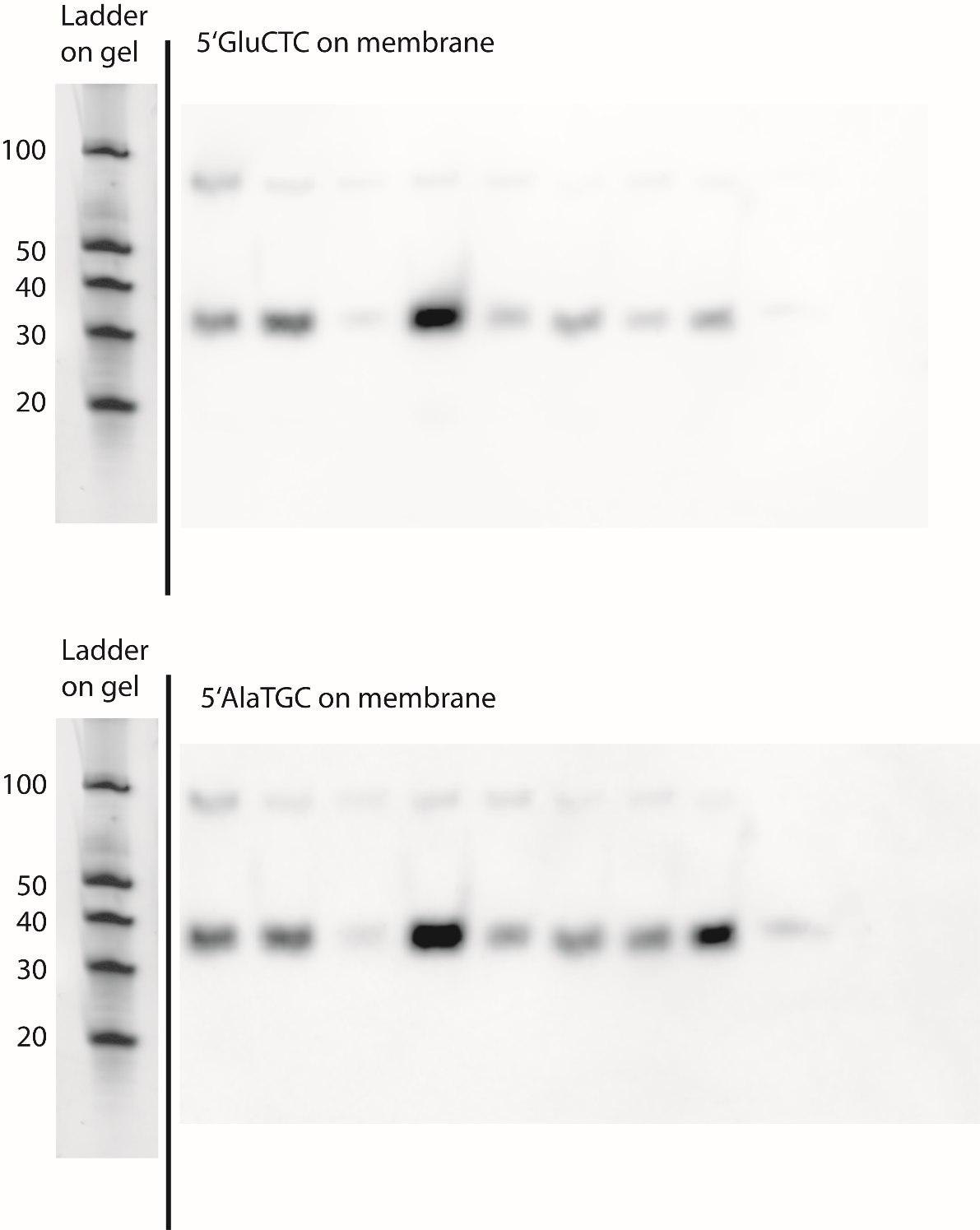


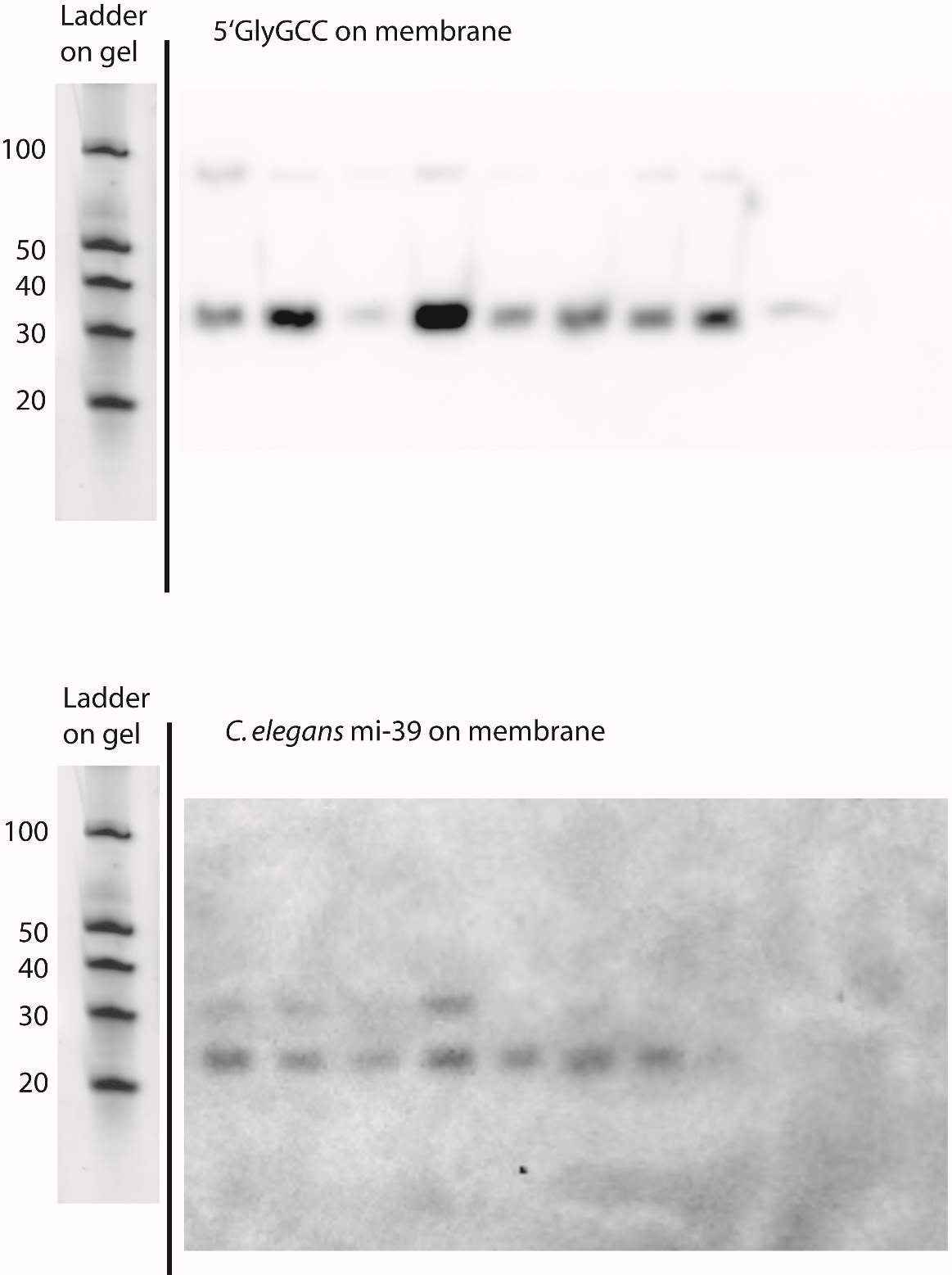

Supplement: Supplementary file 1 — Supplementary Data File. [file 41598_2020_64485_MOESM1_ESM.docx]
